# Framing the Multi‐Centre Qualitative Research Design as a Novel Methodology for Nursing and Healthcare Research: Reflections and A Methodological Discussion

**DOI:** 10.1111/jan.16548

**Published:** 2024-10-24

**Authors:** Jonathan Bayuo, Frances Kam Yuet Wong, Yan Li, Wenze Lu, Arkers Kwan Ching Wong

**Affiliations:** ^1^ School of Nursing, The Hong Kong Polytechnic University Hung Hom Hong Kong

**Keywords:** epistemology, methodology, multi‐centre, nursing, ontology, qualitative

## Abstract

**Aim:**

To discuss the multi‐centre qualitative methodology as a unique design, articulate its guiding paradigm/theoretical perspectives, and highlight its methodological and methodical issues. A secondary objective is to generate further scholarly discourse regarding the multi‐centre approach within the broader qualitative research tradition.

**Design:**

Methodological discussion.

**Findings:**

Rather than an emphasis on only experiences, the multi‐centre approach is presented as a unique design which also focuses on uncovering why a phenomenon or problem exists and perceptions regarding the phenomenon/problem. With its focus on capturing multiple subjective realities, the multi‐centre qualitative design is arguably underpinned by pragmatist constructivism which offers a robust framework for researching phenomenon in a way that is both theoretically informed and practically relevant. Methodologically, the multi‐centre qualitative research design emphasises a problem‐centred enquiry, collaborative approach and rigorous study protocols, systematic site selection, contextual immersion and sensitivity and methodical flexibility.

**Conclusion:**

With the rapidly evolving nursing and global health landscape, the multi‐centre design lends itself to exploring and capturing perceptions on a larger scale compared to single site studies. Careful planning, availability of adequate resources, rigorous protocols and quality assurance plans are critical to ensuring its success.

**Implications for Profession and Patient Care:**

The multi‐centre approach offers the possibility of undertaking the same study across multiple settings/locations which has the potential to improve representation and strengthen transferability.

**Impact:**

This methodological discussion offers clarity regarding the use of the multi‐centre approach and offering strategies for its subsequent uptake in nursing and healthcare research.

**Reporting Method:**

Not applicable.

**Patient and Public Contribution:**

No patient or public contribution.

**Summary**

- What does this paper contribute to the wider global clinical community?
  - This paper discusses the nature of the multi‐centre approach and highlights strategies to improve its conduct.

## 1 Introduction

Qualitative research designs generally focus on uncovering and understanding human experiences. Rather than testing hypotheses or introducing interventions to determine their effects, qualitative research aims to capture and interpret first‐ or second‐order perspectives of the world as lived, perceived or experienced by individuals (Moorley and Cathala 2019). Designs employed within the qualitative research tradition are usually underpinned by varying philosophical orientations such as constructivism and symbolic interactionism which offer assumptions regarding ontology, epistemology and methodology (Duberley, Johnson, and Cassell 2012; Mauthner 2020). Traditionally speaking, the common qualitative research methodologies employed in nursing include phenomenology, grounded theory, ethnography, qualitative description, interpretive description, case study and narrative enquiry (Avgousti 2013). More recently, phenomenography has surfaced as an emerging qualitative methodology for nursing with a theoretical orientation towards a nondualist (relational) ontology and an epistemological stance that emphasises collective knowledge (Bayuo et al. 2024).

The discipline of nursing has long been drawn to qualitative research approaches because they allow researchers to uncover the complexities, subjectivities and social and political contexts of health and illness experiences (Thorne 2009). With its inherent humanistic philosophical foundation emphasising holistic care, nursing aligns seamlessly with the qualitative research tradition (Thorne 2019). Both focus on understanding phenomena in their entirety and capturing the complexity of human experiences. Indeed, qualitative research is considered critical to nursing and healthcare research, as it provides a rich, nuanced understanding of human experiences and interactions, which are essential for delivering compassionate, effective and patient‐centred care.

Despite their notable strengths, such as obtaining rich, in‐depth data, qualitative studies have often been critiqued for being small‐scale, requiring small sample sizes, and limited to single settings, which affects the overall transferability of their findings (Anderson 2010; Khankeh et al. 2015; Lim et al. 2021; Malterud 2001). Besides, due to their emphasis on subjectivity and obtaining rich data, qualitative studies are frequently bound to a specific context and rarely extend beyond the boundaries of a single location (Kapoulas and Mitic 2012; Khankeh et al. 2015; Malterud 2001). These concerns often lead to the notion that qualitative studies are less rigorous and may have a limited place in evidence‐based practice (Given 2006; Pitney et al. 2024; Thorne 2018). As Silverman (p.9) points out, qualitative research is ‘…often treated as a relatively minor methodology [and] it is suggested that it should only be contemplated at early or “exploratory” stages of a study. Viewed from this perspective, qualitative research can be used to familiarise oneself with a setting before the serious sampling and counting begins’ (Silverman 2021). This trend has the potential to marginalise and downplay the value of qualitative research (Crumley and Koufogiannakis 2002; Given 2006).

Thorne has argued that many of the established and conventional qualitative methodologies that the discipline of nursing has inherited from the social sciences have been designed to establish strong theorising rather than to support the very different kind of complex thinking that is needed for excellent nursing practice (Thorne 2016). Indeed, qualitative research is often considered anecdotal and insufficient to make population‐level summaries (Anderson 2010) as well as attain reliability and validity in their truest sense (Agius 2013). Though these perceptions are gradually improving, qualitative research is still viewed as lacking the rigour that is evident in quantitative research (Agius 2013; Ochieng 2009).

To address these concerns, numerous strategies have been proposed. For example, Polit and Beck (2010) have suggested that the replication of studies in multiple contexts and the undertaking of meta‐syntheses of multiple qualitative studies to strengthen evidence base. Given the time‐consuming nature of planning and executing qualitative studies, replicating single studies across multiple contexts can be a daunting task which may not fit well with existing funding priorities. Instead, it may be more practical to consider multi‐centre qualitative studies that examine the same phenomenon across varied settings within the same timeline (Das 2022). Compared to single‐site qualitative studies, a multi‐centre approach confers the benefits of increased representation, obtaining varied perspectives, and strengthening transferability (Das 2022). Also, its emphasis on capturing multiple perspectives offers an opportunity to actively engage with patients and the public as key stakeholders in shaping research and implementing evidence. Thus, the multi‐centre qualitative approach has the potential of generating more comprehensive findings strengthening patient public improvement and engagement (PPIE), ultimately leading to conclusions that may be more conclusive.

## 2 Background

With the rapidly evolving healthcare landscape and similar disease profiles emerging across the globe, both nuanced and shared perspectives are essential to inform and transform practice. Capturing these shared perspectives whilst also accounting for nuanced perceptions across varying contexts is the goal of the multi‐centre qualitative methodology. Studies employing the multi‐centre qualitative methodology focus on recruiting participants and collecting data from multiple institutions within the same or outside a defined geographical area (Das 2022). It is a pragmatic approach to accumulate sufficient numbers of diverse participants than could be attained in a single‐centre study (Lim et al. 2021). Arguably, the multi‐centre approach can facilitate an interdisciplinary, collaborative approach which can enhance group reflexivity and triangulation of results by researchers from diverse backgrounds (Lim et al. 2021). By encompassing multiple contexts and plurality of perspectives, however, these studies introduce a level of complexity that challenges the traditional theoretical orientations and existing perspectives of qualitative methodologies, which are typically suited for single settings.

Further to the above, confusion still exists in existing literature regarding its place within the broader qualitative research tradition. In fact, some studies have utilised the multi‐centre approach within specific qualitative methodologies such as phenomenology (Alkaissi et al. 2022; Belar et al. 2024) and grounded theory (Gonzalo et al. 2013) to highlight repeated data collection across multiple settings. Though this may suggest the use of the multi‐centre approach as a method, other studies have also presented the same approach as the methodology employed (Busetto et al. 2022; Costa et al. 2014; Dhaliwal et al. 2017; Unger et al. 2021). The confusion and highlighted methodological tension make it difficult to appreciate the uniqueness of the multi‐centre approach. More worrying is the fact that different labels have been used to represent this design: ‘multicenter qualitative study’ (Busetto et al. 2022; Costa et al. 2014; Dhaliwal et al. 2017; Unger et al. 2021), ‘multicentric qualitative study’ (Sarabia‐Cobo et al. 2022; Sneyers et al. 2014; Tricou et al. 2022), ‘qualitative descriptive’ (Asmaningrum and Tsai 2018; Facchinetti et al. 2021; Hesselink, Branje, and Zegers 2023; Zhang et al. 2024), ‘qualitative methodology’ (Calderón et al. 2011), ‘cross‐sectional qualitative design’ (Park et al. 2013; Silver et al. 2023) or simply, a ‘qualitative study’ (Adu‐Bonsaffoh et al. 2022; Atif et al. 2021; Chernick et al. 2023; Katz et al. 2023). These indicate a lack of consensus regarding its nature denoting the limited attention that has been paid to this unique methodological approach. If considered qualitative approach, then, its guiding paradigm and philosophical or theoretical foundations remain poorly articulated or loosely defined within the broader qualitative research tradition, leaving its methodological and methodical stances unclear.

### 2.1 Multi‐Centre Design as a Qualitative Methodology

A critical examination of studies employing the multi‐centre approach as a qualitative methodology highlight its focus on understanding experiences, why a phenomenon or problem exists, and perceptions regarding the phenomenon or problem (Atif, Lorcy, and Dubé 2019; Bayuo et al. 2024; Johnson et al. 2012; Lim et al. 2021). Thus, multi‐centre qualitative studies are likely to appear exploratory and descriptive albeit with interpretive hues (Bayuo and Kyei Baffour 2024). These features may help to distinguish the multi‐centre qualitative methodology from other qualitative methodologies. That is, the multi‐centre qualitative approach as a distinct methodology is not concerned with theory development regarding social processes (grounded theory), meaning of an experience as lived (phenomenology) or cultural phenomenon (ethnography) but instead focuses on uncovering participants' perceptions and understandings. Its focus on uncovering perceptions may suggest that the multi‐centre qualitative methodology seeks to capture second‐order perspectives (the world as perceived) whereas several traditional qualitative methodologies emphasise first‐order perspectives (the world as experienced) (Bayuo et al. 2024). This assertion situates the approach as a unique methodology within the broader qualitative research tradition.

However, unlike the other well‐established qualitative methodologies such as grounded theory, ethnography, and phenomenology that come with specific methodological and methodical approaches, there is a notable absence of these for multi‐centre studies. This leaves the burden on researchers to choose as deemed necessary and though this suggests some form of methodical flexibility, it can arguably have significant implications for the study's trustworthiness or methodological rigour. That is, how do we judge the quality of a study in the absence of explicit guidelines fit for the design employed? Key concepts such as bracketing and rigour which are considered critical in the traditional qualitative methodologies are often not explicitly addressed in some studies that have claimed to use a multi‐centre qualitative approach (Atif et al. 2021; Sarti et al. 2018). Whereas established qualitative methodologies such as grounded theory, narrative enquiry, ethnography, and phenomenology focus on the world as ‘experienced’, the multi‐centre qualitative design seem to emphasise the world as ‘experienced and perceived’ similar to the phenomenographic stance (Bayuo et al. 2024). Consequently, the outcomes of studies employing each approach will differ: the more established methodologies will yield a deeper illumination of participants' experiences whereas the multi‐centre approach will produce an understanding of participants' perceptions.

To demonstrate the assertions highlighted in the preceding paragraph, one study that employed a multi‐centre approach to examine how hospitals in the United States approached the prevention of hospital acquired urinary tract infection revealed that clinicians perceived the need for clinical action by including ‘committed advocates/champions’ to promote the removal of unnecessary urinary catheters (Saint et al. 2008). When the phenomenological lens is applied to the experiences of persons living with urinary tract infections, the authors uncovered the meaning of that experience as lived (Solheim 2008; Wilde 1999). In applying the grounded theory approach to middle‐aged and older adults living with urinary incontinence, a substantive theory emerged to facilitate healthcare professionals' comprehension of proactive health behaviours and to understand the mechanisms and manifestations of these behaviours (Zhang et al. 2024).

Prima facie, multi‐centre qualitative studies appear like an extension of qualitative description considering its pragmatist (practical) nature and the focus on description though interpretive hues may be evident (Sandelowski 2000). However, closer theoretical and methodological examination suggest that whereas the outcome of qualitative description is a straightforward description of phenomena (Sandelowski 2000), the multi‐centre qualitative approach with its focus on diverse contexts offers a broader, and potentially more nuanced understanding of perceptions regarding a phenomenon; highlighting overarching themes as well as site‐specific variations (Bayuo and Kyei Baffour 2024).

### 2.2 Articulating the Theoretical/Methodological Gaps and Our Work

The variations highlighted so far suggest that the guiding paradigms, philosophical perspectives and methodologies of the traditional qualitative approaches may not be entirely applicable to underpin the conduct of multi‐centre studies. Thus, there is a great need for philosophical, methodological, and methodical clarity in this regard. The highlighted theoretical/philosophical and methodological tensions/gaps became a focal point for the research team when some members undertook a cross‐country study in Ghana and mainland China (Bayuo et al. 2024). The study aimed to examine the perceptions of adult burn survivors and burn care staff regarding their transition from the burn unit. Given the identified similarities in burn injury epidemiology, clinical management, and limited aftercare support across both settings, the authors argued that it was possible to identify shared concerns that could enhance our understanding of the transitioning experiences. This understanding could inform the development of contextually relevant transitional rehabilitation programs that could be adapted in other settings facing similar post‐burn rehabilitation challenges (Bayuo et al. 2024).

Choosing an appropriate qualitative research design that supported a cross‐country focus proved to be methodologically challenging. Although the authors considered using interpretive description due to its focus on generating knowledge that contributes to clinical practice, they encountered significant methodological tensions and practical considerations, particularly in areas such as sampling and data saturation. Reflecting on their experiences and the theoretical/methodological gaps identified in the literature, a great need to articulate explicitly the philosophical underpinnings and methodology of the multi‐centre approach was noted. We note although the notion of ‘multi‐center’ can be applied as only reflecting data collection from multiple sites, we present it as a distinct methodology rather than a merely a method. To this end, this methodological paper discusses the multi‐centre qualitative research approach within an appropriate philosophical orientation. This will be achieved by articulating its guiding paradigm and theoretical perspectives (ontology and epistemology), methodological and methodical choices, and the practicalities of the design. A secondary goal is to generate further scholarly discourse regarding the multi‐centre qualitative approach within the broader qualitative tradition.

## 3 Theoretical/Philosophical Perspective and Orientation

The multi‐centre qualitative approach emphasises the notion of socially constructed multiple realities across different contexts and cultures. These realities are constructed through interactions rather than discovered and are considered subjective which may suggest leaning towards constructivism or a constructivist philosophical orientation (Peck and Mummery 2018). The focus on capturing shared realities across multiple contexts shaped through interactions indicates co‐construction of realities (Bignold and Su 2013). Considering the focus on capturing co‐constructed multiple realities across settings and a need to adapt the methods to varying contexts also suggests that pragmatism is a potential theoretical/philosophical orientation (Legg and Hookway 2008). Fitting the multi‐centre qualitative approach within the constructivist orientation will help to uncover understandings, insights, and perceptions whereas with pragmatism, we expect methodical flexibility to facilitate the conduct of the study.

Given the potential of employing a multi‐method approach in multi‐centre qualitative studies to uncover multiple subjective realities (rather than objective) suggests that a middle ground between constructivism and pragmatism may be warranted. Thus, pragmatic constructivism is presented as a potential philosophical orientation for multi‐centre qualitative studies (Haas and Haas 2009). By integrating the practical focus of pragmatism with the co‐constructive nature of constructivism, pragmatic constructivism offers a robust theoretical framework for understanding and researching phenomena in a flexible way that is theoretically informed. This approach aligns well with multi‐centre qualitative studies given the focus on capturing multiple co‐created realities across contexts. The pragmatic constructivist stance helps to differentiate the multi‐centre qualitative methodology from other pragmatist‐informed methodologies such as participatory action research (objective and subjective realities directing actions) which lead to actionable/practical outcomes (Greenwood 2007; Kindon, Pain, and Kesby 2007).

### 3.1 Ontology

The ontological features of pragmatic constructivism which underpin multi‐centre qualitative studies include a dynamic/relational and process‐oriented reality, contextualism, and plural/multiple co‐created perspectives (Frankel Pratt 2016). From the pragmatic constructivist lens, reality is considered relational, dynamic, constructed, and contextual which aligns with the understanding that different settings or contexts may have unique realities shaped by their local conditions, cultures, and practices (Haack 1977). Apart from the unique contextual realities, pragmatic constructivism also emphasises the notion of shared realities co‐constructed across settings (Ivanova, Ryabinina, and Tyunin 2019). Reality from this ontological stance is viewed as ever‐evolving and process‐oriented rather than static and fixed. Reality is not seen as a ‘pre‐given entity’ but as something that is continuously shaped and reshaped through human actions and interactions (Krägeloh 2006). This perspective aligns with the idea that reality is not independent of human experiences but is co‐constructed through practical engagements with the world rather than discovered (Krägeloh 2006). Also, this ontological stance focuses on reflective, second‐order perspectives to capture the world not only as experienced, but also as perceived by the social actors therein (Rosiek 2013).

Pragmatic constructivism emphasises the importance of context in shaping reality. The meaning and significance of objects, events, and experiences are understood in relation to their specific contexts (Pihlström 2009). This contextual approach means that what is considered real or true can vary depending on the circumstances in a given situation. Additionally, pragmatic constructivism embraces pluralism, recognising that there are multiple ways of understanding and interpreting the world (Pihlström 2009). From this ontological stance, pragmatic constructivism argues that different perspectives and experiences contribute to a richer and more nuanced understanding of reality (Rosiek 2013). This pluralistic view acknowledges that different contexts and situations may reveal different aspects of reality, and no single perspective can capture the entirety of the truth. That is, there is no single truth out there to be captured. This is essential in multi‐centre qualitative studies wherein diverse viewpoints and perceptions are expected, valued, captured, and reported.

### 3.2 Epistemology

Epistemologically, multi‐centre qualitative studies consider knowledge as subjective. Knowledge is viewed as actively constructed and not passively received from the environment (Olssen 1995). It is not seen as an objective truth but as a provisional and context‐dependent. Knowledge is co‐constructed by individuals through their interactions and shared experiences. This process involves negotiation, dialogue, and collaboration among various stakeholders to co‐create or co‐construct.

Further to the above, the epistemological stance of pragmatic constructivism embraces fallibilism, the idea that all knowledge is provisional and subject to revision (Martela 2015). No belief or theory is immune to doubt or change, and all claims to knowledge are open to scrutiny and improvement (Schwartz 2016). This fallibilistic approach encourages continuous enquiry and adaptation, recognising that our understanding of the world is always evolving (Martela 2015). Enquiry from the pragmatic constructivist lens is considered a process of understanding with a focus on subjective realities. Pragmatic constructivism sees enquiry as an active and iterative process. The iterative and adaptive nature of this philosophical stance is particularly useful in multi‐centre studies where researchers may need to adapt their methods and approaches based on the evolving understanding of each centre's unique context (Misak 2011).

The epistemological stance further emphasises the inter‐subjectivity and communal nature of the such scholarly enquiry (social and communal aspects of knowledge) reflecting a collaborative and participatory approach (Bingham 2004). Knowledge, therefore, is not an individual endeavour undertaken in isolation but is co‐constructed through interactions and dialogue within a community of enquiry. The inter‐subjective nature highlights the importance of communication, collaboration, and shared understanding in the development of knowledge (Bingham 2004). In multi‐centre qualitative studies, this means engaging with participants and stakeholders from different centres to co‐create knowledge that is meaningful and applicable across various settings.

Another key feature of the design's epistemological stance is the nature of truth. For this approach, truth is not an absolute or correspondence with an objective reality but is defined in terms of the practical success of ideas. A belief is considered true if it works effectively in practice and leads to satisfactory outcomes. This conception of truth focuses on the functional and experiential aspects of beliefs rather than their correspondence to an independent reality. Put together, the epistemological stance emphasises knowledge as subjective, subject to revision, generated through interactions, and co‐constructed within a community of enquiry. This philosophical framework encourages a flexible, adaptive, and collaborative approach to understanding and engaging with the world.

## 4 Methodology

Although there are no existing methodological guidelines to employ the multi‐centre approach, it is possible to identify common strands in existing studies to deduce key steps. With the key feature of including more than one setting, it is critical to plan adequately and ensure availability of sufficient resources to employ this design (Lim et al. 2021). Based on our previous work (Bayuo et al. 2024) and gleaning from existing literature, we present the following for consideration in undertaking multi‐centre qualitative studies:

1. ***Problem‐centred enquiry***: With a focus on uncovering why a phenomenon or problem exists and perceptions regarding the phenomenon or problem, a starting point for the multi‐centre qualitative methodology is a gap or problem (Atif, Lorcy, and Dubé 2019; Bayuo et al. 2024; Johnson et al. 2012; Lim et al. 2021). In our previous work, we used informal discussions with clinical staff and burn survivors at the participating settings to ascertain their concerns regarding post‐burn aftercare support (Bayuo et al. 2024). Through the informal discussions, we noticed how language was used to describe similar challenges in Ghana and China. As the informal discussions progressed, we were able to unpack the contextual similarities and variations that had shaped the problem. In the end, we uncovered it was the same practice gap/challenge, that is, poor aftercare support for burn survivors, albeit with different names or labels.
2. ***Collaborative approach***: Congruent with the pragmatic constructivist stance, early engagement with stakeholders, practitioners, patients, and communities is extremely important as they must consider the identified issue as a problem requiring attention. Establishing a team with research representation from each participating site will be helpful. A steering committee or central monitoring team with representation across all participating settings can also be considered (Johnson et al. 2012; Lim et al. 2021). Unlike participatory action research that emphasises a collaborative approach throughout the research process, in the multi‐centre design this may or may not be that extensive such as actively involving research participants in data analysis.
3. ***Contextual immersion and sensitivity***: With the strong emphasis on relational and contextual reality, being immersed in the participating settings and being sensitive are critical. Such contextual immersion and sensitivity are important to interpret the experiences/perspectives using the context as a frame of reference. Thus, the focus is not a mere straightforward description of their experiences but a presentation of their experiences/perspectives in light of their contexts generating both nuanced and shared findings. In our study, two research team members were present at the burn centre in China and one member had previously worked in the burn centre in Ghana. Data collection and analysis were undertaken by these persons independently with ongoing wider team consultation. During data analysis process, they were able to employ their understanding of each context to interpret the data following which subsequent team meetings focused on merging the findings and isolating both shared and nuanced concerns across the two settings. Shared concerns included the presence of post‐burn residual needs and the need for cost‐effective aftercare support emerged. Nuanced findings regarding the nature of existing peri‐discharge support emerged as comprising patient education in Ghana and reminders regarding follow‐up surgical interventions in China (Bayuo et al. 2024).
4. ***Methodical flexibility***: Flexibility regarding the choice of methods is one of the cornerstones for undertaking multi‐centre qualitative studies which is congruent with the pragmatic constructivist stance. With the inclusion of more than one context or undertaking a study across more than one setting, it is possible the data collection methods that worked in one context may not work in another context requiring a methodological/methodical shift. The focus on capturing multiple realities rather than a single, absolute truth offers researchers the opportunity of employing diverse methods (McArdle 2022). This allows researchers to be open and adaptable to changes within the research congruent with the pragmatist constructivist stance. For instance, semi‐structured may be appropriate for one setting whereas in another, focus group discussions may be more helpful considering the nature of the context and practical issues. In our study, semi‐structured interviews were used at both study sites since it was feasible to undertake interviews rather than group discussions. In one study, however, both interviews and focus group discussions were employed to obtain data (Lim et al. 2021). Other approaches to qualitative data collection such as observation and document/diary reviews, are equally applicable.

## 5 Methods: Sampling, Sample Size, and Data Saturation

Qualitative methodologies such as phenomenology emphasises the inclusion of only participants with the lived experience under exploration albeit the multi‐centre approach can include diverse stakeholders in one study. Similar to other qualitative approaches, purposive, convenience, snowball, or theoretical sampling approaches may be considered appropriate for a multi‐centre qualitive study (Rai and Thapa 2015). Purposive sampling approach will help to maximise variability within the sample based on identified characteristics such as professional group, rank/professional level, experience, and status (Sneyers et al. 2014). Convenience sampling, where practicable, may also be considered but should be employed in such a way to allow for representation of participating stakeholders from each context. Both purposive and convenience sampling approaches will require researchers to select the participant sampling criteria prior to conducting research (Shorten and Moorley 2014). Congruent with the notion of pluralism, however, some multi‐centre qualitative studies have employed the theoretical sampling approach which is not bounded by the limits of a priori selection and instead, focuses on jointly collecting and analysing data to decide what data to collect next and where to find the participants (Ellegaard et al. 2018; Johnston et al. 2014). Placing one approach above the other may be inappropriate considering the flexibility associated with this design. However, for practical reasons, determining the sample characteristics a priori may help to identify potential stakeholders to engage/collaborate early in the study. If there is a need to recruit more as the study unfolds, this can be done since the pragmatist philosophical stance permits such flexibility. Thus, regardless which approach a research team decides to use, there should be clear justifications in relation to that particular multi‐centre study.

Determining the sample size for multi‐centre qualitative studies remains a contentious area in existing methodological literature. Two trends have been identified: either determine the sample size based on saturation at each participating site independently (Bayuo et al. 2024) or a priori sample size determination which is then stratified across the participating sites (Calderón et al. 2011; Chernick et al. 2023). Although the former approach can help to achieve a thick description from each context (Freeman 2014; Jenks 2002), it can lead to large datasets which could be challenging to manage. Besides, it may appear as though undertaking multiple studies which could blur the lines between a case study and a multi‐centre qualitative study. Considering the pragmatist constructivist stance, choosing one approach over the other may not be helpful. Instead, research teams should consider what works better considering the nature of the study, the number and nature of contexts involved, characteristics of the potential participants and resources available. In existing multi‐centre qualitative studies, reported sample sizes include 21 (Sneyers et al. 2014), 34 (Gonzalo et al. 2013), 35 (Roten et al. 2022), 36 (Jones et al. 2021), 44 (Sinuff et al. 2007), and 46 (Bayuo et al. 2024). Data saturation across these studies was considered the point where no new findings were identified similar to single centre qualitative studies.

With the great need for pluralism in multi‐centre qualitative studies, sample size may also be guided by information power, and not just data saturation (Malterud, Siersma, and Guassora 2016). Determining information power is governed by the study aim, sample specificity, use of an established theory, quality of dialogue, and analytical strategy (Malterud, Siersma, and Guassora 2016). A broad study aim as usually occurs in multi‐centre qualitative studies necessitates a larger sample size to provide adequate information power, as the phenomenon being investigated is more extensive. Conversely, a smaller sample may suffice if the participants possess characteristics that are highly specific to the study aim, compared to a sample with participants of less specificity (Malterud, Siersma, and Guassora 2016).

Sample size for focus group discussions may vary compared to individual interviews. Although there is currently no guidance regarding sample size for focus group discussions for multi‐centre qualitative studies, single centre studies suggest between 4 to 8 groups (Guest, Namey, and McKenna 2017; Hennink and Kaiser 2022). In one multi‐centre study, the authors employed 5 focus group discussions comprising of 5–13 members (Baraff et al. 1992). In another study, 10 focus groups were constituted (Casassa et al. 2021). Considering the practical issues that may arise in multi‐centre studies, quoting an exact figure may be methodologically incongruent. Instead, the reported sample sizes should be used as examples to guide and inform other studies which can be adapted based on the unique circumstances and contexts.

## 6 Methods: Data Collection Approaches

With a pragmatic constructivist theoretical underpinning, diverse qualitative data collection approaches that can help to obtain subjective data can be employed. Existing studies reporting the use of the multi‐centre qualitative approach have reported the use of a blend of data collection approaches such as interviews, focus group discussion, and observations (Bayuo et al. 2024; Gonzalo et al. 2013; Johnston et al. 2014; Scott et al. 2018). As a qualitative design, other approaches that can be considered include documents, diaries, and artefacts if related to the phenomenon under investigation. Interviews, if employed, should emphasise a semi‐structured approach to gain an in‐depth understanding of the respondent's perspectives whilst also allowing for flexibility to adapt to emerging issues as they study proceeds. Also, the fallibilistic epistemological stance suggests that more than a single episode of interview or discussion may be needed.

If interview or group discussion is considered appropriate for a multi‐centre, the interview/topic guide must be piloted at each participating sites to offer an opportunity to refine it further for language accuracy as pertains to each context before the main data collection. The piloting period can help to strengthen the rapport and further open discussion for the study. The participants should be given the opportunity to discuss freely based on the questions asked and probing questions to elicit further in‐depth information can be used (Majid et al. 2017). Field notes, where practicable, should be obtained as the interview proceeds. All interviews should be audio recorded with participants' permission to facilitate the data analytical process. Where focus group discussions are utilised, existing guidelines regarding the constitution and conduct equally applies (Curtis and Redmond 2007; Doody, Slevin, and Taggart 2013; Jayasekara 2012). Additionally, the interviews or focus group discussions should be tailored towards uncovering perceptions, insights, and understandings. Data collection should occur concurrently with data analysis to guide the refinement of the topic guide, finalisation, and justification of sample size. Also, it is essential that personnel assigned for data collection purposes be well immersed in that context.

Language barriers may be evident in multi‐centre qualitative studies that cross borders. In one multi‐centre project which was undertaken across Italy, the Netherlands, Poland, United Kingdom, Spain, and Sweden, the authors reported using five languages across the participating sites (Johnson et al. 2012). To facilitate reporting and analysis, it will be helpful for the research to be undertaken in the local language and a common language considered by the team for reporting and interpreting the findings.

Regarding the timing of data collection, it is possible to consider either concurrent data collection across the participating sites particularly if they are in the same location. However, in the instance where participating sites are separated by geographic distance, it may be challenging to observe daily work on an ongoing basis and may be challenging to concurrent data collection. Most likely, one site may be ahead of another (Reynolds et al. 2011). Research teams should therefore consider which approach may work better in their unique circumstances.

## 7 Methods: Data Management and Analysis

With the pragmatist stance, the multi‐centre qualitative methodology supports the employment of computer‐assisted data management using relevant software. As with other qualitative methodologies, all interview or focus group recordings should be transcribed verbatim noting non‐verbal cues. As highlighted in the preceding section, all interviews/group discussions must be undertaken in the local language and transcribed verbatim in the local language at the initial phase. Transcription can be done manually by a team member who is natively fluent in the language used for the interview or discussion. With the rise in technological applications, an appropriate transcription software can be employed for transcription (Bayuo et al. 2024). However, in this instance, the output must be checked for accuracy independently by team members before proceeding to the subsequent analytical stages (Bayuo et al. 2024).

Following transcription, the next critical step will be to translate the transcribed data into a common language agreed by the research team. In our case, this was English which required the transcripts in simplified Chinese to be translated to English. Translation must be done by a native speaker and reviewed by team members who are fluent in both the original and translated versions. To ensure rigour, the translated transcripts can be discussed with some participants to ensure meanings are retained. Once consensus has been achieved that the translated transcripts are a true reflection of the interviews/discussions, the formal analysis can begin.

Existing studies employing the multi‐centre methodology have reported the use of a plethora of data analysis approaches including thematic analysis (Bayuo et al. 2024; Hughes et al. 2020; Jones et al. 2021; Saint et al. 2008; Zhang et al. 2024), content analysis (Costa et al. 2014; Johnson et al. 2017), and constant comparison approach (Cook et al. 2009; Gonzalo et al. 2013; Johnston et al. 2014; Scott et al. 2018; Sinuff et al. 2007; Tricou et al. 2022). Regardless which analytical approach is used, the process should seek to capture the multiple ways of understanding or perceiving the phenomenon under exploration.

We recommend the use of analytical strategies highlighted by Johnson et al. (2012) presented as Table 1. This focuses on segmenting data according to the participating sites, creating a codebook, ascertaining the reliability by independent coders, and modifying the codebook (Johnson et al. 2012). With the focus on context, it is important to group participants based on their setting and analysis undertaken independently in these groups before mixing to capture the wider group perspectives. In this way, both shared and nuanced findings will be unpacked. In presenting the study findings, both shared and nuanced perceptions should be evident in the exemplars presented.

**TABLE 1:** Strategies to consider during data analysis.

No.	Points to consider
1.	Researchers at each participating site to be immersed in the data to generate a list of narrative codes. This will help to segment the data based on each participating site.
2.	The emerging narrative codes from each site should be shared and reviewed with the wider research team to develop a draft codebook.
3.	Emerging codes from each site should be recorded separately as the team works identifying similar codes across settings.
4.	Similar codes across settings should be assembled into groups and a descriptive label assigned. Attention should be paid to nuanced codes unique to a specific site.
5.	Team consensus/agreement should be achieved regarding the groups of similar codes to be aggregated into the codebook.
6.	Team agreement should be attained regarding the meaning of the English translation of the developed codes before progressing the analysis in the native language of the participating settings.
7.	Aggregate groups of similar codes to formulate categories.

## 8 Methods: Methodological Rigour/Trustworthiness

Considering the nature of multi‐centre qualitative studies and its underpinning theoretical stance, methodological rigour or trustworthiness will not be interested in discovering a single truth or reality. Instead, it will be about capturing both nuanced and shared perceptions and understandings shaped by local policies, cultures, and resources and the generation of knowledge in response to the identified gap. Also, the diversity across the participating settings should be evident (Jackson, Halcomb, and Walthall 2023). Some studies have reported using Lincoln and Guba's framework regarding credibility, transferability, dependability, and confirmability (Bayuo et al. 2024; Facchinetti et al. 2021). In addition to this framework, a quality assurance plan will be needed to ensure trustworthiness (Johnson et al. 2012). The quality assurance plan should ensure that researchers carefully document the methods and protocols and provide consistent methods across participating sites (Johnson et al. 2012). A standardised template can be developed and used by the research teams to report on their adherence to established quality assurance strategies formulated by the group.

Considering the nature of the study, a detailed description of all research steps/processes and the contexts involved should be captured. This is particularly essential as the approach offers methodological and methodical flexibility requiring researchers to clearly document and justify the methodological choices in relation to the study. There should be evidence of contextual immersion and sensitivity to attain trustworthiness. Data analysis should be undertaken by team members who has been immersed in a particular context to use their understanding as a frame of reference for the analysis.

With the notion of co‐creation in multi‐centre studies, the role of bracketing remains unclear though in one study, the authors mentioned that they noted their pre‐conceptions regarding the phenomenon under investigation (Facchinetti et al. 2021). Thus, to advance the voice of the study participants and maximise the co‐creation process, the researchers' preconceived ideas should be noted and considered during the data collection and analytical process. The study findings should be discussed with the study participants/stakeholders to enable understanding of the actionable insights obtained. This can be carried out by convening a discussion with the stakeholders to present and discuss the findings in relation to the problem.

## 9 Ethical Considerations

Navigating ethics can appear daunting for a multi‐centre qualitative study. In our previous work, we obtained ethics approval at three levels: firstly, from our academic institution and then separately from each participating centre which was due to the inclusion of sites from two different jurisdictions. Such an approach may be time consuming as one has to be completed before the other. However, for studies undertaken across multiple sites in the same country, a single approval from the relevant authority may be sufficient. Regardless, we suggest that researchers employing this design allow for sufficient time to complete all ethical application requirements. A steering committee, if constituted to support the study should review the study in an ongoing manner to capture emerging ethical issues from members in each participating site and work actively to resolve these. Quality control and monitoring are essential to the success of multi‐centre qualitative studies and to ensure adherence/compliance to relevant regulations.

## 10 Discussion

With the rapidly evolving healthcare landscape across the globe, multi‐centre qualitative studies lend themselves to exploring and uncovering perceptions, insights, and understandings. Compared to single‐centre studies, the multi‐centre approach can offer greater utility, increased representativeness, overcome issues associated with generalisability, and greater explanatory power (Das 2022). Though the conduct of multi‐centre qualitative studies is not a new phenomenon, its theoretical orientation, methodological, and methodical concerns have received little to no attention which can have significant implications for quality and methodological rigour/trustworthiness (Das 2022). This methodological paper therefore situates itself to address the identified gap and to generate further scholarly discourse. We present the theoretical/philosophical orientation of the multi‐centre qualitative design to inform its methodology and methods. As these propositions are used, we hope there will be ongoing scholarly discussions and avenues for further refinement to ground the multi‐centre qualitative approach in its rightful place within the broader qualitative research tradition.

With its theoretical basis underpinned by the pragmatic constructivist stance, the multi‐centre qualitative approach seeks to uncover multiple co‐constructed subjective realities across more than one setting. This is distinct and unique when compared to the more traditional, well‐established qualitative methodologies. For instance, by employing a phenomenological approach, an in‐depth understanding of lived experiences can be uncovered from the pre‐reflective first‐order perspective. The multi‐centre approach will however help to understand phenomena from the second‐order perspective. Thus, multi‐centre studies may have a significant place in the discipline of nursing/healthcare research by improving representativeness and generating both shared and nuanced findings. The emergence of public health issues such as COVID‐19 disrupted the delivery of healthcare services across the globe requiring all systems to adjust within a short time to meet the increasing needs of patients (De Benedictis et al. 2022). Though the impact varied across settings globally, common strands regarding its impact are visible such as the experience of burnout and compassion fatigue among frontline nurses (Billings et al. 2021; Ding et al. 2022; Nikbakht Nasrabadi et al. 2022). Such a major issue requires working collectively rather than individually which lends support to the use of multi‐centre studies to identify actionable insights in curbing this global menace. As globalisation proceeds further, the healthcare landscape and disease profiles are also evolving with much similarities across settings than previously perceived (Walt 1998). Thus, now more than ever is a great need to break silos of single site studies and to consider a multi‐centre approach to generate findings that are locally/contextually relevant and internationally applicable.

Nursing has long been attracted to qualitative methodologies. The intersection of nursing with multi‐centre qualitative studies enriches the research process and outcomes, leading to improved patient care, enhanced professional collaboration, and the development of evidence‐based practices that are applicable across diverse settings (De Chesnay 2014). By leveraging the strengths of multiple institutions and capturing a wide range of experiences and perspectives, these studies provide a comprehensive understanding of complex phenomena (Das 2022). Also, the ability to gather data from a diverse range of patient populations by including multiple centres will enable researchers to capture a wide array of experiences and perspectives, which is crucial for understanding the varied needs of patients. This diversity ensures that the findings are more comprehensive with stronger transferability and providing insights that are applicable to a broader population (Lim et al. 2021). The collaborative spirit ingrained in multi‐centre qualitative studies not only advances the field of nursing but also helps to build a supportive professional community (Johnson et al. 2012; Priest et al. 2007; Ulrich et al. 2015).

Undertaking a single‐site qualitative study in itself can be time consuming. Thus, a multi‐centre study will require careful planning and allocation of adequate resources (Lim et al. 2021). The notion of methodical flexibility comes in handy to enable researchers to choose methods considered to be contextually appropriate rather than remaining faithful to specific methodologies. Conducting qualitative research across multiple centres can potentially allow for the pooling of resources, including funding, personnel, and other resources (Das 2022). This resource sharing can enhance the quality of the research and make it more feasible to conduct large‐scale studies (Das 2022). By leveraging the strengths and capabilities of multiple settings, researchers can undertake more ambitious projects that would be difficult to accomplish independently. What is more, findings from multi‐centre qualitative studies can have significant implications for nursing policies and practices. By providing evidence from multiple settings, these studies can inform policy decisions and lead to improvements in nursing education, practice, and administration. Policymakers can use the insights gained from these studies to develop guidelines and regulations that promote best practices and enhance patient care.

The multi‐centre qualitative approach is not without limitations. With its focus on uncovering insights, it is possible to argue that it is not suited for every type of qualitative research question. For instance, if the goal of a research is to understand a phenomenon or experience, other qualitative methodologies may be more appropriate than the multi‐centre approach. Thus, its use should be congruent with the research question and aim. The notion that reality is dynamic implies that the insights/perceptions generated today may be outdated tomorrow indicating a need to constantly update what is known today in keeping with the pragmatist stance. Issues regarding logistical and operational complexities, budgetary constraints, and strategies to ensure effective communication and collaboration should be considered early in a project employing this approach as they can impact the conduct of the study.

## 11 Conclusion

As we conclude this discussion on the methodology of the multi‐centre qualitative design, it is evident that whilst our methodological discussion has provided valuable insights, there are still numerous avenues for further work. Future studies should aim to address the challenges of coordinating and standardising qualitative data collection across multiple sites and improving consistency. Additionally, expanding the scope to include a more diverse range of centres and participant demographics could enhance the comprehensiveness of findings. We also recommend exploring the integration of advanced data analysis techniques and digital tools to streamline the synthesis of qualitative data from various centres. By fostering collaboration among researchers and institutions, and by continuing to refine and innovate our methodological approaches, we can deepen our understanding and improve the rigour of multi‐centre qualitative research. We call upon the academic community to engage in this ongoing dialogue and contribute to the evolution of best practices in this vital area of study designs.

## Ethics Statement

The authors have nothing to report.

## Conflicts of Interest

The authors declare no conflicts of interest.

## Peer Review

The peer review history for this article is available at https://www.webofscience.com/api/gateway/wos/peer‐review/10.1111/jan.16548.

## Data Availability

Data sharing is not applicable to this article as no new data were created or analysed in this study.
